# Supplementary material for: Tribological Properties of Nitrate Graphite Foils
Source: Nanomaterials (Basel). 2024 Sep 15;14(18):1499. doi: 10.3390/nano14181499 (PMC11434555; doi:10.3390/nano14181499)
Supplement: Supplementary file 1 [file nanomaterials-14-01499-s001.zip › X-ray data/misorientation/0001/over500/dens_16/TD/210.pdf]

# Raw data

| | | | | | |
|---|---|---|---|---|---|
| Sample name: | | X-ray | : Cu / 40 kV / 30 mA | Counter | : Scintillation counter |
| File | : 210.raw | Goniometer | : Ultima IV | | |
| Comment | : | Attachment | : MPA-ML4(with gamma) for stress | | |
| Date | : May-16-24 12:29:49 | Filter | : K-beta filter | Scan mode | : Continuous |
| Operator | : Administrator | I.Monochro | : CBO | Scan speed | : 5.00 deg./min. |
| | | C.Monochro | : Not installed | Sampling width | : 0.05 no unit |
| | | DivSlit | : Open | Scan axis | : theta |
| | | DivH.L.Slit | : 10.00mm | Scan range | : 10.00 -> 80.00 no unit |
| | | SctSlit | : 2/3?‹\|2/3deg. | Fixed angle | : 0.00 no unit |
| | | RecSlit | : 0.30mm | | |

Memo :

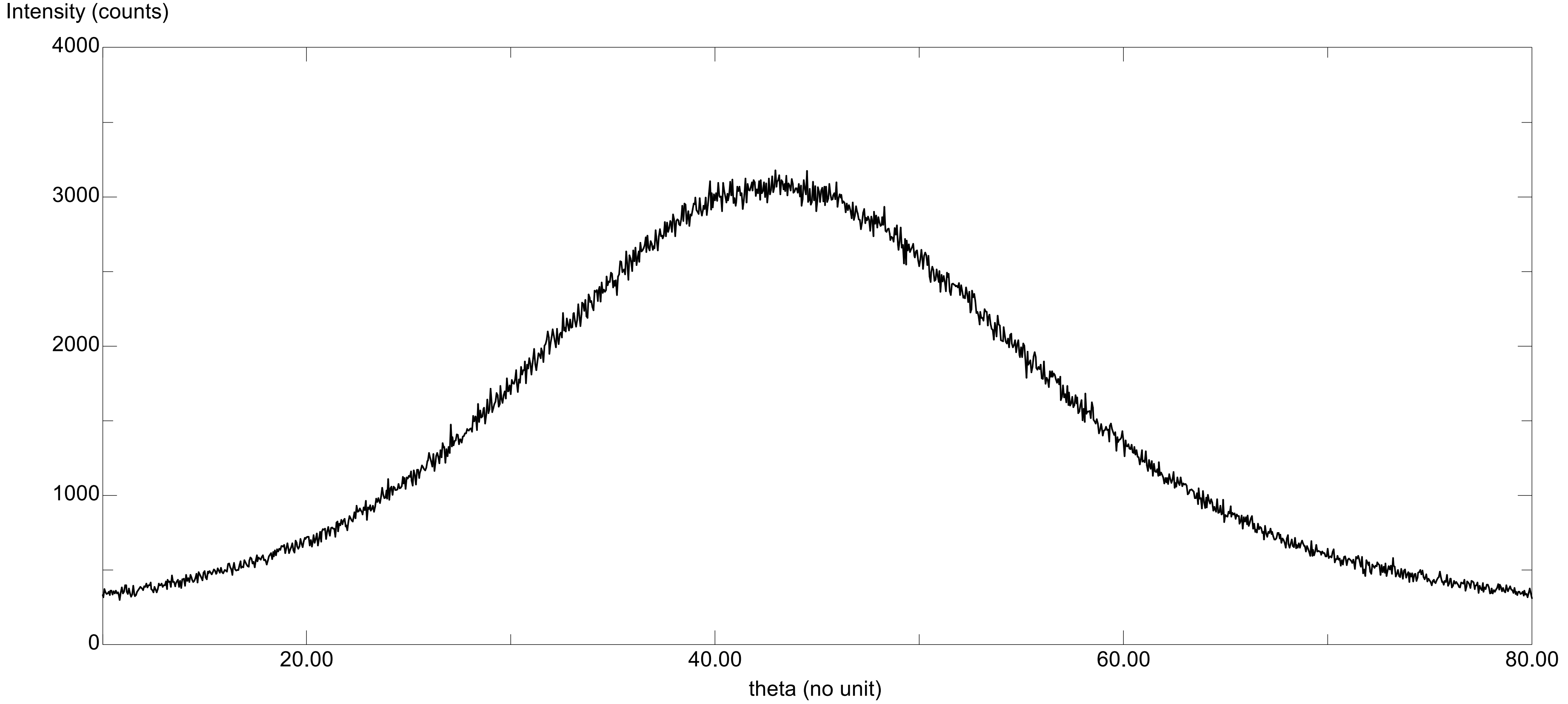

# Integral Int. Calculation ( History )

Sample name: File : 210.raw Date : May-16-24 12:29:49 Operator : Administrator

Comment : Memo :

Peak calc. method: FW2/3M

| h | k | l | Start | Stop | Peak | d-value | FWHM | Max. int. | | Gross int. | Integ.w | Integ. int. |
|---|---|---|---|---|---|---|---|---|---|---|---|---|
| 0 | 0 | 0 | 10.00 | no unit80.00 | no unit43.23 | no unit2.09 | 27.35 | no unit2719 | counts | 1583677.00 | 29.123 | 79183.85 |

[ Cut ]
[ Slit Correction ]
[ LP Corr. ]

[ Absorp. Corr. ]
[ Norm. ]
[ Smoothing ] Method : Averaging Smoothing Points: 11
[ BG subtraction ] Method : Averaging at the end
Low angle : 5 High angle : 12
[ Ka2 elimination ]

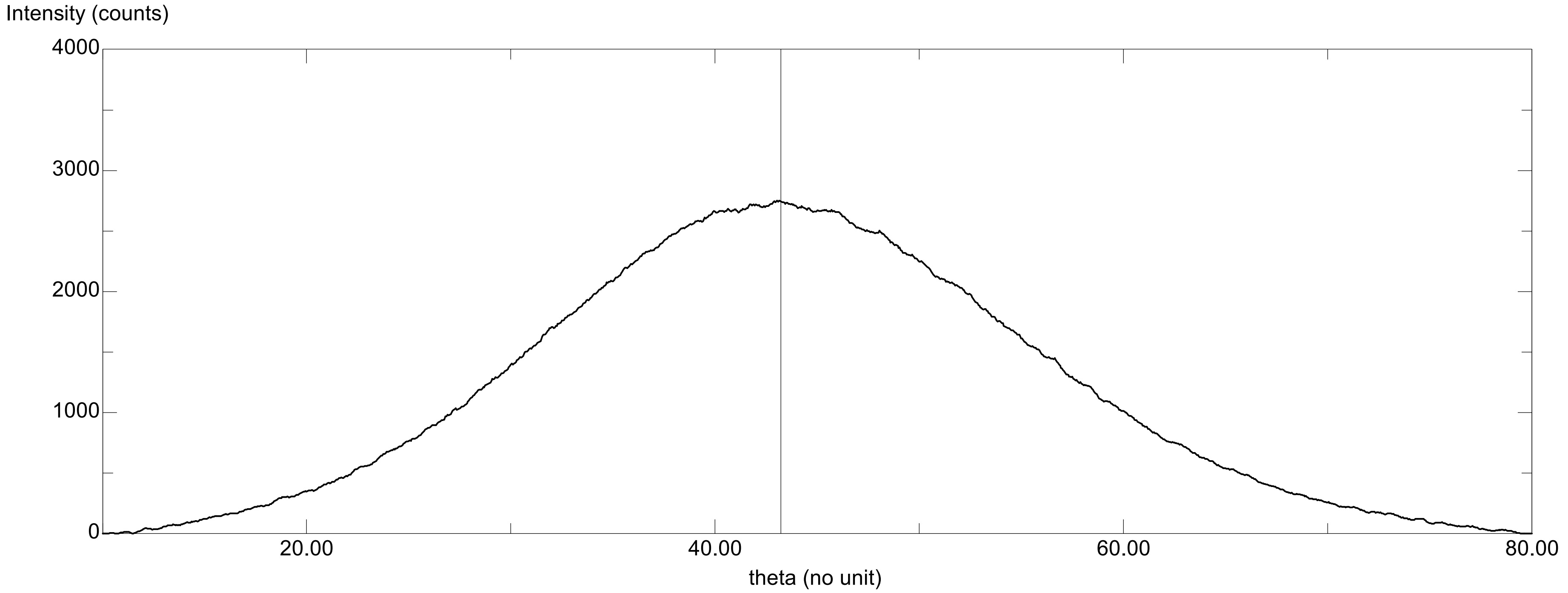

# Integral int. sum table

Sample name: File : 210.raw Date : May-16-24 12:29:49 Operator : Administrator
Memo :
Peak calc. method: FW2/3M

| No. | h | k | l | Start | Stop | Peak | d-value | FWHM | Max. int. | Gross int. | Integ.w | Integ. int. | Comment |
|---|---|---|---|---|---|---|---|---|---|---|---|---|---|
| 1 | 0 | 0 | 0 | 10.00 | 80.00 | 43.23 | 2.09 | 27.35 | 2719 | 1583677.00 | 29.123 | 79183.85 | |
